# Glycine is able to induce both a motility speed in- and decrease during zebrafish neuronal migration

**DOI:** 10.1080/19420889.2018.1493324

**Published:** 2018-08-13

**Authors:** Ulrike Theisen, Sven Hey, Christian D. Hennig, Ralf Schnabel, Reinhard W. Köster

**Affiliations:** a TU Braunschweig, Zoological Institute, Cellular and Molecular Neurobiology, Braunschweig, Germany; b TU Braunschweig, Institute for Genetics, Braunschweig, Germany

**Keywords:** Neuronal migration, neurotransmitters, glycine, glycine receptor alpha 1, NKCC1, zebrafish

## Abstract

Various neurotransmitters influence neuronal migration in the developing zebrafish hindbrain. Migrating tegmental hindbrain nuclei neurons (THNs) are governed by depolarizing neurotransmitters (acetylcholine and glutamate), and glycine. In mature neurons, glycine binds to its receptor to hyperpolarize cells. This effect depends on the co-expression of the solute carrier KCC2. Immature precursors, however, typically express NKCC1 instead of KCC2, leading to membrane depolarization upon glycine receptor activation. As neuronal migration occurs in neurons after leaving the cell cycle and before terminal differentiation, we hypothesized that the switch from NKCC1 to KCC2 expression could alter the effect of glycine on THN migration. We tested this notion using in vivo cell tracking, overexpression of glycine receptor mutations and whole mount in situ hybridization. We summarize our findings in a speculative model, combining developmental age, glycine receptor strength and solute carrier expression to describe the effect of glycine on the migration of THNs.

## Introduction

Neuronal migration is an essential developmental process contributing to the formation of a functional brain. It is thought that migration underlies the correct positioning of interacting neuronal partners, which is the basis of circuitry [1,2]. Several external factors control migrating neurons, such as chemical and mechanical guidance cues. Recently, we have reported that neurotransmitters influence the migratory speed of tegmental hindbrain nuclei neurons (THNs) in live zebrafish embryos [3]. THNs emerge from a primordial zone, the upper rhombic lip (URL), to cross the developing cerebellum in a first phase to reach the midbrain-hindbrain-boundary (MHB) [4,5]. In a second migratory phase, the THNs follow the MHB ventrally to form clusters. Later in development, these neuronal clusters form the nucleus isthmi, the secondary gustatory/viscero-sensory nucleus, and the superior reticular nucleus [4].

In our model, derived from timelapse imaging studies of zebrafish embryos, neurotransmitters exhibit regionally restricted influences on the migratory speed, rather than directionality. Such zones could arise as a result of changing protein expression patterns along route: THNs in phase 1 have recently left the cell cycle, while THNs at the end of migration have initiated differentiation, as indicated by the formation of axons [6]. This implies that some receptors, co-factors or downstream molecules may be expressed during specific periods of differentiation, for example only become expressed at a later stage in migration. In the case of glycine, this notion could apply to the expression of solute carriers. Proliferating neuronal precursors and early differentiating neurons typically express NKCC1 (*slc12a2*) [7–9]. This increases the intracellular Cl^−^ concentration, which leads to Cl^−^ efflux upon glycine receptor opening, and consequently membrane depolarization [7,10]. It is thought that regular depolarization via glycine and NKCC1 is necessary to maintain the proliferation of neuronal precursors [11–13]. The hyperpolarizing effect of glycine in mature neurons in contrast [14,15] depends on the co-expression of KCC2 (*slc12a5b*) [15,16]. KCC2 lowers the intracellular Cl^−^ concentration, which causes an influx of Cl^−^ opening of the glycine receptor, and membrane hyperpolarization. Weak expression of this solute carrier begins in THNs when the cells have reached the ventral portion of the MHB in > 28 hpf embryos [3].

Here we test the effects of glycine on THN migration in younger embryos and modulate it with mutations affecting glycine receptor strength. We propose that NKCC1 is active in early-migrating THNs, and that NKCC1 may play a role in regulating late-migrating THNs when glycine receptor signaling is over-active.

## Results and discussion

**Figure 1.**
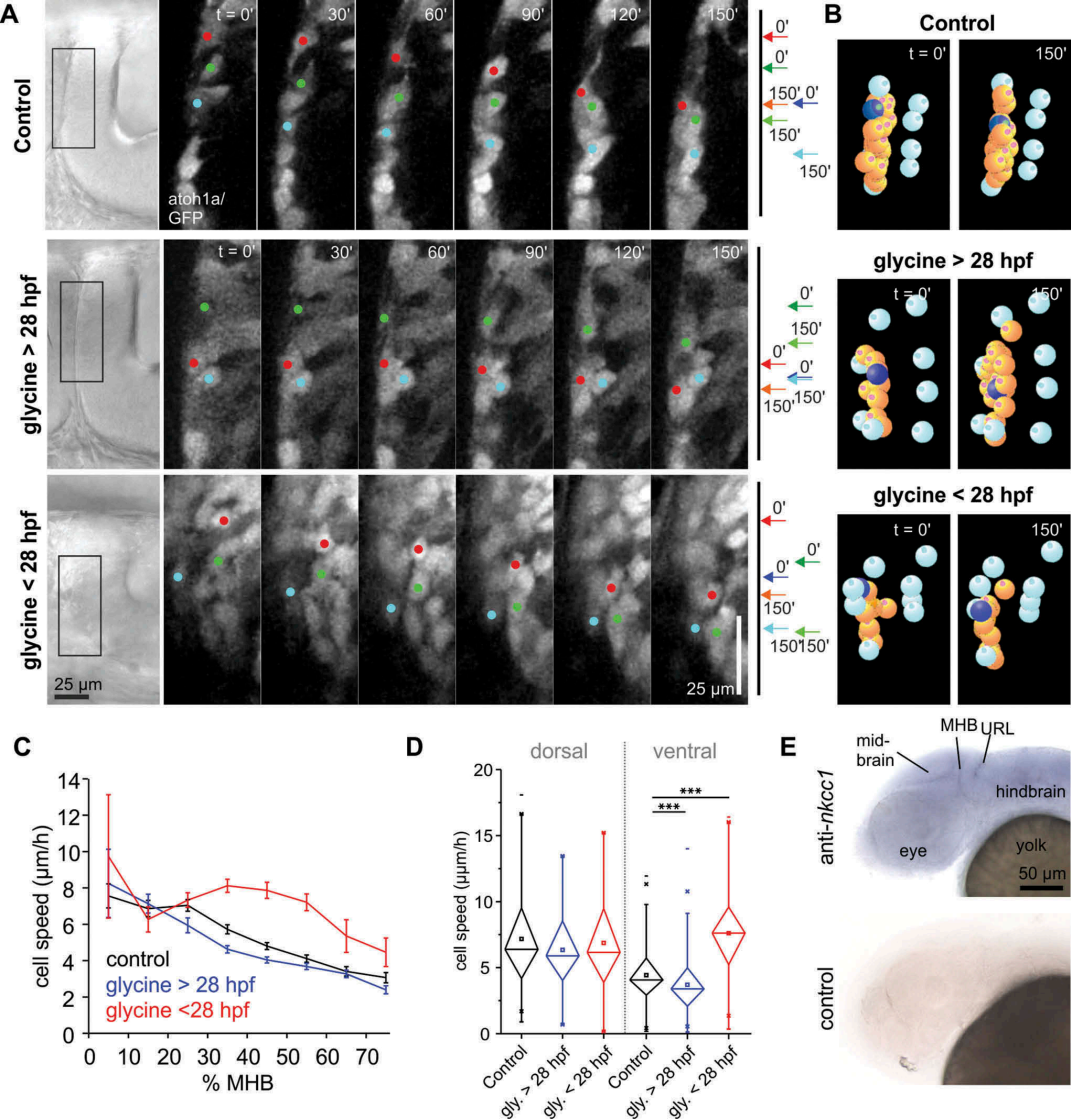
Glycine de- or increases THN speed depending on embryo age. **(a)** Adding 10 mM glycine to embryos produces a THN speed increase in 24 hpf embryos (bottom panel), while in 30 hpf embryos induces a speed decrease. Images for control and > 28 hpf embryos are taken from [3]. Boxes on the transmitted light images on the left indicate the region magnified in the stills from the fluorescent films. Colored dots indicate the position of example cells, also represented by arrows on the right hand side pointing out the start and end positions of the same examples. Scale bars: 25 µm. Elapsed time is given at the top. See also Video 1. **(b)** THNs were tracked in SIMI°BioCell and tracks corrected for tissue shifts using markers represented by cyan-colored bowls. THNs are shown in orange, and one example marked in blue. See also Video 2. **(c)** Excess glycine in young embryos increases THN speeds in the central region of the MHB, comparable to the region where excess glycine slows THNs in older embryos. Tracks were classed by their starting point in 10% bins along the MHB, where 0% is the dorsal, 100% the ventral end of the MHB. Values for control and glycine > 28 hpf are taken from [3]. **(d)** Statistic analysis confirms the notion that glycine affects THNs most strongly in the ventral part of the MHB, using 0–25% for dorsally, and 32.5%-100% for ventrally localized cells as cutoff. Error bars represent SEM. Values for control and glycine > 28 hpf are taken from [3]. **(E)** WISH analysis against *nkcc1 (slc12a2)* demonstrates that the gene is widely expressed in the head and brain of the embryo at 30 hpf.

In our previous study, we observed that excess glycine produces a mild decrease in THN speed in cells migrating in phase 2 along the MHB [3]. The study was carried out in embryos > 28 hpf, as most THNs migrate at this developmental stage. Yet a few phase 2 THNs can already be found at the MHB in 24 hpf embryos. The addition of 10 mM glycine to 24 hpf embryos increased THN speed, which contrasts the speed decrease observed in older embryos (Figure 1(a–d), Table 1, Videos 1 and 2). As immature neuronal precursors typically express NKCC1 leading to depolarization upon exposure to glycine [7], and the hyperpolarization-mediating KCC2 is only expressed in THNs > 28 hpf [3], NKCC1 activity could explain this effect. Whole mount in situ hybridization analysis (WISH) confirmed that the gene is widely expressed in the head of 27 – 30 hpf embryos (Figure 1(e)), similar to results from [17].

**Table 1.** THN track analysis statistic values. Values for Control (DMSO) and glycine on > 28 hpf embryos are taken from [3].

Condition	N (embryos)	N (tracks)	p value (to control)	Chi^2^ (to control)
Control (DMSO) dorsal	19	147		
Control (DMSO) ventral	19	356		
Control + Bumetanide dorsal	12	102	0.71645	0.13192
Control + Bumetanide ventral	12	184	0.24062	1.37693
Glycine > 28 hpf dorsal	20	72	0.21939	1.50835
Glycine > 28 hpf ventral	20	609	5.7123 × 10^−8^	29.45863
Glycine < 28 hpf dorsal	15	58	0.60204	0.27193
Glycine < 28 hpf ventral	15	170	1.989 × 10^−27^	111.72773
GlyRa1 I67F dorsal	18	34	0.48558	0.48631
GlyRa1 I67F ventral	18	174	1.0835 × 10^−4^	14.9854
GlyRa1 V304M dorsal	29	58	0.02543	4.9946
GlyRa1 V304M ventral	29	251	0.12619	2.33878
GlyRa1 V304M + Bumetanide dorsal	18	84	0.42238 (to V304M: 0.00926)	0.64368 (to V304M: 6.77131)
GlyRa1 V304M + Bumetanide ventral	18	161	0.82367	0.04965
GlyRa3 P185L dorsal	19	56	0.01567	5.83959
GlyRa3 P185L ventral	19	194	0.29574	1.0933

**Figure 2.**
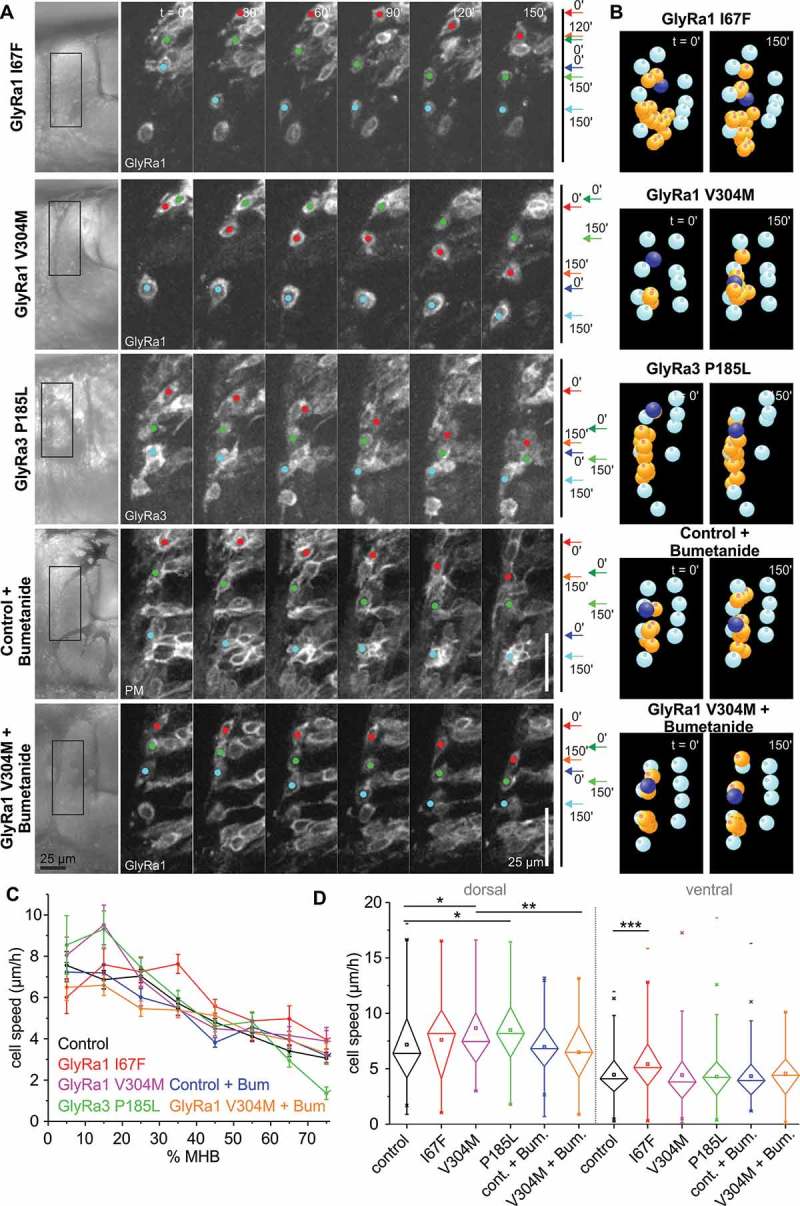
Gain-of-function mutations in glycine receptors lead to increased cell speed. **(a)** In contrast to the overexpression of wt GlyRa1 receptor (see [3]), the overexpression of gain-of-function mutations in GlyRa1 or GlyRa3 produce THN speed increases. The addition of Bumentanide counteracts this effect. Boxes on the images on the left indicate the magnified region. Colored dots indicate the position of examples, also shown by arrows on the right hand side. Scale bars: 25 µm. Elapsed time is given at the top. See also Videos 3 and 5. **(b)** After track correction and quantification, THNs are shown in orange, and one example marked in blue. See also Videos 4 and 6. **(c)** Interestingly, after classing tracks by their starting point in 10% bins along the MHB, it appears that mutations produce their effects either in the dorsal or the ventral region. Values for control are taken from [3]. **(d)** Statistic analysis confirms the regional influence of glycine receptor mutations. The effect of GlyRa1 V304M disappears upon Bumetanide treatment. Error bars represent SEM. Values for control are taken from [3].

The results prompted us to further test the function of glycine receptors in THN migration. The overexpression of wt glycine receptor a1 in > 28 hpf embryos led to a mild speed decrease (approx. 20% reduction) compared to a loss-of-function control (Y226F; see [3]). This is consistent with the beginning expression of KCC2, leading to a hyperpolarizing effect of glycine in these cells. We aimed to increase the speed reduction effect by overexpressing gain-of-function mutations in glycine receptors. The mutations are modelled on available human patient data, and correspond to zebrafish GlyRa1 I67F [18,19], GlyRa1 V304M [18,20,21], and rat GlyRa3 P185L [22]. In contrast to expectations, all of these mutations led to a moderate THN speed increase in phase 2 (approx. 20–25% increase; Figure 2(a–d), Table 1, Videos 3 and 4). GlyRa1 V304M and GlyRa3 P185L affect mostly dorsally-located THNs, while GlyRa1 I67F appears to act mostly on ventrally localized THNs. As the dorsal region of the MHB is the area where many THNs which have recently left the cell cycle arrive at the MHB, we speculated that the effect of these mutations could be the result of retained NKCC1 activity. If so, glycine should induce a strong depolarization in these cells, and thus increase speed. Hence, we added 100 µM Bumetanide, a NKCC1 inhibitor, to the medium, which suppresses the speed increasing effect of GlyRa1 V304M (Figure 2(a–d), Table 1, Videos 5 and 6).

**Figure 3.**
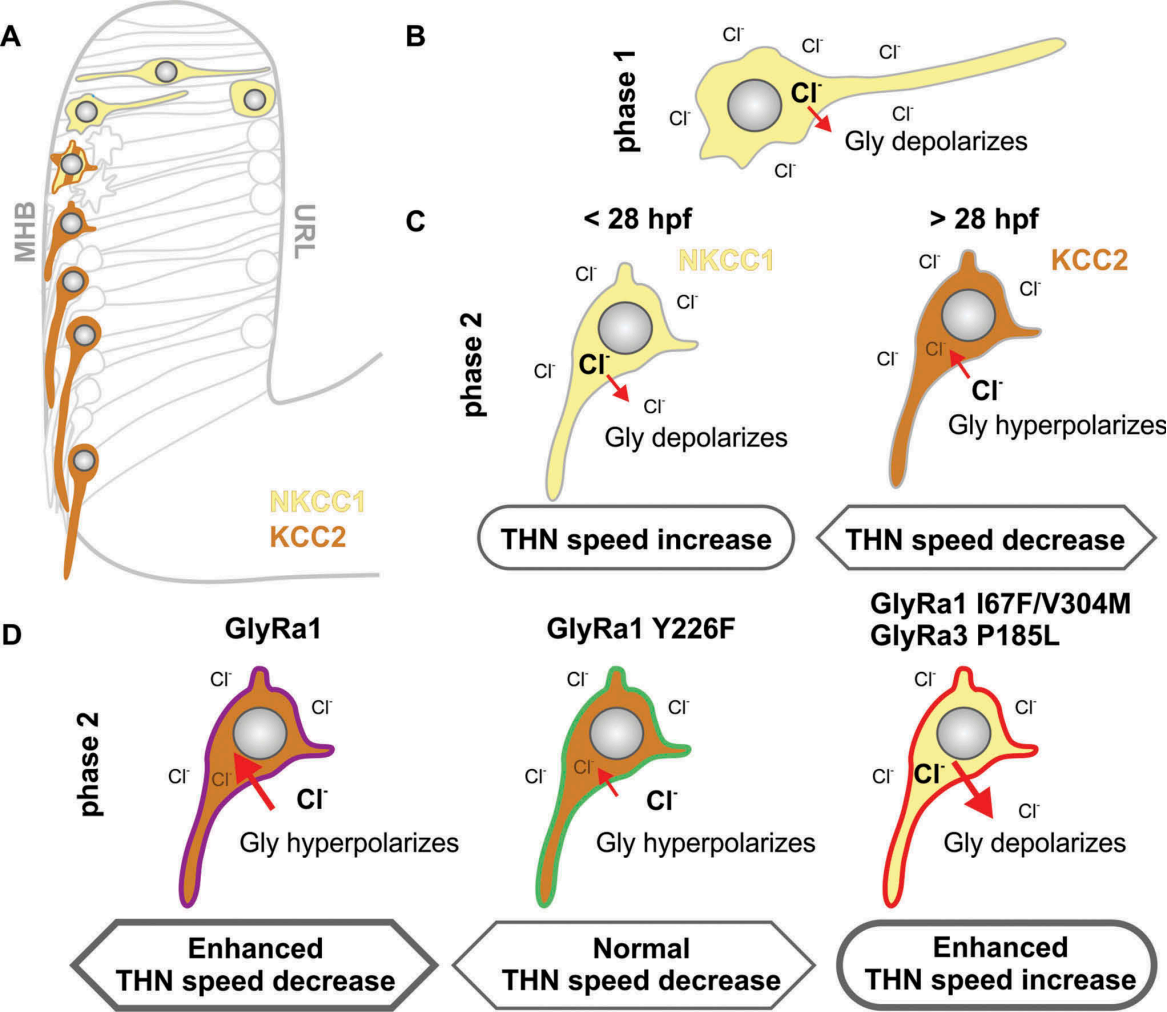
Model showing how NKCC1/KCC2 co-expression with different glycine receptor strength can regulate THN speed. **(a)** THNs in phase 1 express NKCC1 (yellow), while KCC2 (brown) becomes expressed only in differentiating phase 2 THNs. **(b)** The expression of NKCC1 leads to high concentrations of intracellular Cl^−^, so that normal glycine receptor channel opening produces a Cl^−^ efflux, and effective depolarization. **(c)** In THNs which express KCC2, however, the intracellular Cl^−^ concentration is maintained at low levels. When the glycine receptor is opened upon glycine binding, Cl^−^ enters the cell to produce hyperpolarization, and an overall speed decrease. As the expression of KCC2 could only be detected from > 28 hpf [3], the wide-spread expression of NKCC1 in younger embryos could explain the speed increase observed upon glycine treatment in younger vs older embryos. **(d)** The overexpression of GlyRa1 wt or loss-of-function mutation Y226F does not affect this normal expression pattern of NKCC1/KCC2, hence produces an increase in the hyperpolarization in wt overexpression, or no change in the loss-of-function mutant. In case of an overexpression of the gain-of-function mutations, the observed increased THN speed likely depends on the persistent expression of NKCC1, potentially as a survival mechanism against increased Cl^−^ influx.

These findings indicate that NKCC1 and KCC2 underlie the opposing responses to glycine in zebrafish THNs, similar to the situation described in mammalian models [7,15]. In this speculative model, newly emerging THNs inherit NKCC1 from precursor cells and therefore exhibit higher intracellular Cl^−^ levels (Figure 3(a)). The opening of the glycine receptor facilitates an efflux of Cl^−^ and membrane depolarization ([7,23], Figure 3(b)), increasing THN speed. In early migrating THNs in young embryos, the presence of NKCC1 persists into phase 2 migration at the MHB, still promoting THN speed (Figure 3(c)). From > 28 hpf onwards, KCC2 is expressed. This reduces the intracellular Cl^−^ concentration, so that glycine receptor opening results in an influx of Cl^−^ from the extracellular space ([15], Figure 3(c)), which leads to hyperpolarization, and THN speed decrease.

Based on this postulated normal response to glycine, we speculate that some glycine receptor mutants may alter this glycine response pattern in > 28 hpf embryos. The overexpression of wt GlyRa1 in our scenario is expected to increase the sensitivity of THNs to endogenous glycine and consequently increased hyperpolarization and speed reduction due to the action of KCC2. A loss of function mutation, which is unable to bind glycine, does not show any altered response to glycine in overexpression, as long as endogenous receptors are available (Figure 3(d)). Gain-of-function mutations, however, appear to promote the activity of NKCC1 over KCC2 at this older developmental stage (Figure 3(d)), as indicated by the inhibitor data. Although the molecular details of such a connection between glycine receptor strength and altered functionality of NKCC1/KCC2 remain to be characterized, it has been reported that glycine receptor activity and solute carrier function correlate in late differentiating neurons [24], and a shift in solute carrier expression and activity patterns upon brain lesions or neuropathy has also been reported [25–29].

Future work using in vivo reporters for the expression of NKCC1 and KCC2 and inhibitor studies addressing the functionality of the solute carriers will elucidate the interactions between the solute carriers and the glycine receptor, and how this connection influences neuronal migration.

## Methods

All procedures involving animals were carried out according to EU guidelines and German legislation (EU Directive 2010_63, license number AZ 325.1.53/56.1-TU-BS). All experiments were carried out using fertilized eggs from the fish line Tg(atoh1a:Gal4TA4) (line hzm2Tg) [6], crosses of Tg(atoh1a:Gal4TA4) with Tg(4xUAS-GFP) (hzm3Tg) [30], or wt brass.

All methods including animal handling, microinjection, THN imaging, tracking and statistics have been described in [3]. Significance levels were tested by Kruskal-Wallis-ANOVA and p-values represented as p < 0.05 (one star), p < 0.01 (two stars), p < 0.001 (three stars), see also Table 1. The DIG-containing probe for NKCC1 was generated by in vitro transcription of 1 kb sequence in the 3ʹ half of the gene. WISH analysis was carried out as described previously on PFA-fixed embryos [3]. Plasmids encoding for glycine receptor mutations were generated by point mutation based on the full-length wt described in [3] and [22]. Bumetanide (Sigma, B3023) was prepared as a 20 mM stock solution in DMSO and used at 100 µM dilution in 30% Danieau medium. Images were adjusted using ImageJ, SIMI Bio°Cell and Photoshop, data analysis and statistic testing in Origin, and figures arranged in CorelDraw.
