# Supplementary material for: Glycine is able to induce both a motility speed in- and decrease during zebrafish neuronal migration
Source: Commun Integr Biol. 2018 Aug 13;11(3):1–7. doi: 10.1080/19420889.2018.1493324 (PMC6132429; doi:10.1080/19420889.2018.1493324)
Supplement: Supplemental Material [file kcib-11-03-1493324-s001.zip › Supplemental material.docx]

**Supplemental material**

**Video Legends:**

**Video 1:** Glycine increases or decreases THN migration speeds, depending on embryonic age. Individual cells are labelled with colored dots. Elapsed time is given in h:min. Scale bar: 25 µm. Control and glycine > 28 hpf embryos are taken from [[3](#_ENREF_3)].

**Video 2:** Track representation of THNs after glycine application. Several examples are shown in orange, with one highlighted (dark blue), after tissue shift correction, with correction markers given in cyan. Elapsed time in h:min. Control and glycine > 28 hpf embryos are taken from [[3](#_ENREF_3)].

**Video 3:** Gain-of-function mutations in the glycine receptor increase THN speed. Individual cells are labelled with colored dots. Elapsed time is given in h:min. Scale bar: 25 µm. Film for GlyRa1 wt is taken from [[3](#_ENREF_3)].

**Video 4:** Track representation of migrating THNs which overexpress gain-of-function mutations in the glycine receptor. Several examples are shown in orange, with one highlighted (dark blue), after tissue shift correction, with correction markers given in cyan. Elapsed time in h:min. Film for GlyRa1 wt is taken from [[3](#_ENREF_3)].

**Video 5:** Application of Bumetanide rescues the THN speed increase caused by GlyRa1 V304M. Individual cells are labelled with colored dots. Elapsed time is given in h:min. Scale bar: 25 µm.

**Video 6:** Track representation of THNs expressing GlyRa1 V304M under the influence of Bumetanide. Several examples are shown in orange, with one highlighted (dark blue), after tissue shift correction, with correction markers given in cyan. Elapsed time in h:min.
